# Differences in serum oxidative status between glaucomatous and nonglaucomatous cataract patients

**DOI:** 10.1186/s12886-017-0409-3

**Published:** 2017-02-15

**Authors:** Wojciech Rokicki, Jolanta Zalejska-Fiolka, Dorota Pojda-Wilczek, Alicja Hampel, Wojciech Majewski, Serap Ogultekin, Ewa Mrukwa-Kominek

**Affiliations:** 1 Department and Clinic of Ophthalmology, School of Medicine in Katowice, Medical University of Silesia, Ceglana 35, 40-514 Katowice, Poland; 2 Department of Biochemistry, School of Medicine with the Division of Dentistry in Zabrze, Medical University of Silesia, Katowice, Poland; 3 Radiotherapy Department, Maria Sklodowska-Curie Memorial Cancer Center and Institute of Oncology, Gliwice Branch, Poland

**Keywords:** Glaucoma, Oxidative stress, Serum, Neurodegenerative disease

## Abstract

**Background:**

Oxidative stress contributes to both intraocular pressure regulation and glaucomatous neuropathy. The systemic redox status (solitary determination) was examined in primary open-angle glaucoma (POAG) patients with cataract and nonglaucomatous cataract patients. Cataract-matched group comparisons appear more precise in the context of oxidative stress evaluation. The aim of this study was to establish if systemic oxidative status in POAG patients was elevated compared with the cataract only subjects.

**Methods:**

The study included patients with primary open angle glaucoma (POAG group, *n* = 30) and controls (non POAG group, *n* = 25). Serum concentration of lipofuscine (LPS), malondialdehyde (MDA) and activity of total superoxide dismutase (SOD), and its mitochondrial (Mn-SOD) and cystolic (Cu,Zn-SOD) isoform were measured. Total oxidant state (TOS) and total antioxidant capacity (TAC) in blood were also evaluated.

**Results:**

Significant increase of LPS (*p* = 0.0002) and MDA (*p* = 0.005) concentration was observed in glaucomatous patients as compared with controls. Total SOD activity was significantly lowered in the glaucoma group (*p* = 0.003); serum level of Mn-SOD was significantly lower in glaucoma patients (*p* = 0.048) however, Cu,Zn-SOD was not. Glaucoma patients presented elevated mean TOS (*p* = 0.016). Both groups presented with comparable TAC.

**Conclusion:**

Systemic redox balance of cataract patients was significantly altered in the course of glaucoma.

## Background

Glaucoma refers to several disorders having the same clinical features. Characterized by progressive retinal ganglion cell (RGC) and axon loss; glaucoma causes damage to the optic nerve and results in gradual visual field loss. Subsequently, this leads to irreversible blindness. Despite well-developed diagnostic tools and relatively efficient treatment, glaucoma still remains the world’s leading cause of irreversible blindness. Glaucomatous neuropathy may progress with elevated or normal (arbitrarily estimated) intraocular pressure (IOP). Therefore, elevated IOP, the main known risk factor for glaucoma, is neither enough nor necessary to trigger glaucomatous neuropathy. Research suggests a multifactorial etiology of glaucoma pathogenesis; nevertheless, the trigger(s) initiating glaucomatous pathology still remains unidentified. Primary glaucoma should not be considered solely as an ocular pathology [1]. Data suggests oxidative stress in glaucomatous disturbances do not conflict with other observations but complements mechanical, vascular, genetic and immunologic theories in the pathogenesis of glaucoma. Oxidative stress presumably plays an important role in increasing IOP, producing trabecular meshwork alterations and promoting neuronal cell death affecting retinal ganglion cells in glaucoma [2, 3]. Furthermore, an increase in IOP is understood to generate oxidative stress in retina [4]. POAG in the context of oxidative stress presents two main front lines of oxidation and defense against oxidative stress. The first front line in the anterior segment functions when UV and visible light are the main resources of exogenous ROS, produced mostly in aqueous humor. The second front line is in the well vascularized posterior segment and functions when endogenous and systematic ROS are delivered with blood [5]. Additionally, there is an age-dependent increase in production of endogenous free radicals and ROS. Therefore, both POAG occurrence and age dependent systemic redox balance deterioration presumably have common pathways.

In this research serum oxidative state represented by oxidative degradation products (malonyl dialdehyde - MDA, lipofuscine - LPS) and selected antioxidant enzymatic defense (total superoxide dismutase - SOD, and its isoenzymes: Mn-SOD and Cu,Zn-SOD) were studied. We also evaluated the oxidant and antioxidant status by measurement of total oxidant status (TOS) and total antioxidant capacity (TAC) respectively.

MDA, widely regarded as a marker of a peroxidative damage to cell membranes, is induced by physical and/or chemical oxidative stress. Nucci et al. demonstrated that glaucoma patients had significantly higher levels of serum and humor aqueous MDA as compared with nonglaucomatous controls [6].

LPS - Lipofuscin (called age pigment) is a marker of normal aging. Lipofuscin tends to accumulate throughout life in post-mitotic cells, such as neurons and glia. Dolman et al. in 1980 reported the presence of lipofuscin in the optic nerve [7]. The subsequent investigations linked LPS accumulation with age-related disorders, especially with POAG [8].

As previously reported, human and animal ocular fluids and tissues contain one of the major antioxidant enzymes - superoxide dismutase (SOD, EC1.15.1.1) which plays a key role in protecting against oxidative damage [9]. SOD activity alteration was previously reported in aqueous humor of glaucoma patients [10].

The aim of this present research was to assess serum oxidative stress in glaucoma/cataract patients compared with cataract only controls.

## Methods

The study protocol was approved by the Ethics Committee of School of Medicine in Katowice, Poland (permission number: KNW/0022/KB1/123/10) and adhered to the tenets of the Declaration of Helsinki for experiments involving human tissue and samples.

### Participants

The POAG group was comprised only of Caucasians. Only patients whose eye was scheduled for antiglaucomatous drainage surgery due to progressive visual field loss and whose target IOP was not reached pharmacologically, was taken under analysis.

Patients with previous history of IOP over 23 mmHg within the last 6 months before examination and on sampling day were excluded. We set the threshold of 23 mmHg, arbitrary (21 mmHg + 10%). We presuppose, IOP ≤ 23 mmHg is not yet in the acute phase of intraocular hypertension, and a limitation of 21 mmHg would reduce our examined group. All patients presented bilateral visual field defect.

The Controls group included Caucasians who were scheduled for cataract surgery.

Sequential inclusion criteria both for POAG and for the Controls group were as follows:

(1) no previous intrabulbar surgery, (2) between 65 and 75 years old, (3) best corrected visual acuity of 0.5 or better (Snellen’s charts) (4) no myopia or hyperopia >3D (dioptres) (5) non-smokers, (6) no documented, diagnosed, treated ophthalmic and organic diseases (only treated arterial hypertension was accepted), (7) no abnormalities in the routine preoperative laboratory tests especially in C-reactive protein (CRP), complete blood count (CBC) and differential, (8) body mass index (BMI) < 30.

Patients for examined groups were chosen, according to above mentioned criteria, and from patients consecutively admitted to The Cataract/Glaucoma Station of The Department of Ophthalmology, Medical University of Silesia for planned surgery.

### Ophthalmic examination

Clinical evaluation of POAG included gonioscopy, detailed ophthalmoscopy, central corneal thickness measurement, tonometry (Goldmann’s, Haag-Streit, Bern, Switzerland; 0.5% Alcaine) visual field examination (Octopus 301 HS, Interzeag) and policlinic history analysis. The average IOP for each patient was determined by three measurements (the day before admission, day of admission and the day of surgery). All IOP measurements were taken during morning hours (between 8 AM and 11 AM). The IOP policlinic history (within the last 6 months) together with our measurements guided us to exclude POAG patients in the acute IOP phase.

The patients were examined on the day of blood sample collection.

### Blood sample collection

Blood samples were collected into chemically clean tubes to obtain serum. After coagulation samples were centrifuged at room temperature for 10 min at 3000 rpm, serum was retracted and transferred into clean tubes for biochemical analysis. Pending analysis, serum samples were frozen at −80 ° C for further studies.

### Electrophysiological Examination

The transient pattern electroretinogram (PERG) was examined using Reti-Port equipment (Roland Consult, Germany). The study conditions were performed as per the recommendations and standards of the ISCEV (International Society for Clinical Electrophysiology of Vision) [11]. Square checks with check size 30’, contrast 97%, reversal rate four reversals per second were used. Two trials for each stimulus condition were obtained to confirm reproducibility, 200 sweeps were collected and averaged. Fiber electrodes as recording electrodes, gold-cup as a reference, and ground electrodes were used. The patients wore best optical correction for the distance of examination (1 m). Implicit time (the time to peak) and amplitude of the negative wave N95 (from the peak of P50 to the trough of N95) were measured.

### Biochemistry

#### Determination of superoxide dismutase activity (SOD, Mn-SOD, Cu,Zn-SOD)

Oyanagui’s method [12] was used to measure the activity of SOD in blood serum. In this method, xanthine oxidase produces superoxide anions, which react with hydroxylamine forming nitric ions. These ions react with naphthalene diamine and sulfanilic acid generating a colored product. Concentration of this product is proportional to the amount of superoxide anions produced and is negatively proportional to the activity of SOD. Absorbance was measured using an automated Perkin Elmer analyzer at a wavelength of 550 nm. The enzymatic activity of SOD was expressed in nitric units. The isoenzymes of SOD, Mn-SOD and CuZn-SOD, were also indicated using KCN as the inhibitor of the CuZn-SOD activity. The activity of SOD is equal to one nitric unit (NU) when it inhibits nitric ion production by 50%. Activities of SOD in blood serum were expressed in NU/ml.

#### Determination of malondialdehyde (MDA) concentration

The product of lipid peroxidation - MDA was measured fluorometrically as 2-thiobarbituric acid-reactive substance (TBARS) in blood serum according to Ohkawa [13] with modifications. Samples were mixed with 8,1% sodium dodecyl sulfate, 20% acetic acid and 0,8% 2-thiobarbituric acid. After vortexing, samples were incubated for 1 h at 950 C and butanol-pyridine 15:1 (v/v) was added. The mixture was shaken for 10 min. and then centrifuged. The butanol- pyridine layer was measured fluorometrically at 552 nm and 515 nm excitation (Perkin Elmer, USA). TBARS values are expressed as malondialdehyde (MDA) equivalents. Tetraethoxypropane was used as the standard. Concentrations are given in μmol/l plasma.

#### Determination of Total Oxidation Status (TOS)

Total oxidant status was measured according to Erel [14] in blood serum. The assay is based on the oxidation of ferrous ion to ferric ion in the presence of various oxidant species in acidic medium. The change in color of the ferric ion by xylenol orange was measured as a change in absorbance at 560 nm. This process was applied to an automated Perkin Elmer analyzer and calibrated with hydrogen peroxide. Data is shown in μmol/l.

#### Determination of Total Antioxidant Capacity (TAC)

Total antioxidant capacity was measured according to Erel [15] in blood serum. In this colorimetric method, radicals are generated and the antioxidant activity of blood serum reduces radical formation. The change in color of ABTS+ ions (2,2′-azinobis(3-ethylbenzothiazoline-6-sulfonate) was measured as the change in absorbance at 660 nm. This method was conducted in an automated Perkin Elmer analyzer calibrated with Trolox. Data is shown in mmol/l.

#### Determination of lipofuscin concentration

In blood serum, the LPS concentration was determined according to Jain [16]. Fluorescence was measured using an LS45 spectrofluorimeter Perkin Elmer at wavelengths of 360 nm (absorbance) and 440 nm (emission). Values are presented as relative units (relative fluorescence lipid extract, RF), where X corresponds to a fluorescence solution of 0.1 mg/mL quinidine sulfate in 0.1 N sulfuric acid.

### Statistical

The statistical analysis was performed with a Statistica package. The comparison between groups was performed either with parametrical *t*-test or with non-parametrical Mann-Whitney test if assumptions of a parametrical test were not met.

## Results

Baseline patient characteristics are summarized in Table 1.

**Table 1:** Patients characteristics

	Glaucoma + Cataract		Vs	Cataract	
Sex (men/female)	♂ = 14 ♀ = 16	*n* = 30	N/S	♂ = 10 ♀ = 15	*n* = 25
Age (years)	68 ± 5,42		*p* = 0.21	69 ± 3,72	
Median duration of known glaucoma (years)	5–12 8,6 ± 3,3		Ø	Ø	
Intraocular pressure (IOP)	21,0 mmHg SD: 2,35 95% CI: 20,12–21,88		*p* = 0.000	16,3 mmHg SD: 1,43 95% CI: 15,69–16,85	
BCVA (best corrected visual acuity)	0,72 SD: 0,18		NS	0,66 SD: 0,16	
N95 amplitude (pattern electroretinography PERG)	2,05 SD: 1,09 95% CI:1,64–2,45		*p* = 0.000	3,3 SD: 1,48 95% CI: 2,70–3,89	
N95 implicit time (pattern electroretinography PERG)	97,93 SD: 9,41 95% CI: 94,42–101,45		NS	97,15 SD: 7,63 95% CI: 94,01–100,24	

Only one eye per patient was included in the study. Number of patients with IOP < =21 was 17 and with IOP > 21 was 13.

No significant differences between groups in BMI was recorded.

Serum concentration of malondialdehyde (MDA), the indicator of lipid peroxidation was significantly raised in the glaucoma group (1.16 μmol/l; SD: 0.54; 95% CI: 0.96–1.36) as compared with controls (0.757 μmol/l; SD: 0.13 95% CI: 0.70–0.81) *p* = 0.005 (Table 2).

**Table 2:** Differences in oxidative stress markers between groups

	Glaucoma + Cataract	Cataract	*p*
Malondialdehyde	1.16 μmol/l SD: 0.54; 95% CI: 0.96–1.36	0.757 μmol/l SD: 0.13 95% CI: 0.70–0.81	*p* = 0.005
Lipofuscine	1636.27 RF SD: 325.03 95% CI: 1514.90–1757.64	1298.84 RF SD: 241.59 95% CI: 1201.26–1396.42	*p* = 0.000
Total SOD^a^	17.48 NU/ml SD: 3,12 95% CI: 16,3–18,6	20,43 NU/ml SD: 4,03 95% CI: 18,80–22,06	*p* = 0.003
Cytosolic SOD^a^	8.87 NU/ml SD: 2.44	9.84 NU/ml SD: 2.20	NS
Mitochondrial SOD^a^	8.61 NU/ml SD: 2.04	10.59 NU/ml SD: 3.49	*p* = 0.048
Total Oxidative State	22.81 μmol/l SD: 26,44; 95% CI: 12,94–32,68	8.08 μmol/l SD: 5,82; 95% CI: 4,71–9,35	*p* = 0.016
Total Antioxidant Capacity	1.03 mmol/l SD: 0,19; 95% CI: 0,96–1,10	1.02 mmol/l SD: 0,09; 95% CI: 0,98–1,06	NS

^a^ *SOD* Superoxide dismutase

The serum lipofuscin varied between compared patients (Table 2). In glaucomatous subjects the mean concentration of “age pigment” was elevated to 1636.27 RF; SD: 325.03 95% CI: 1514.90–1757.64 while nonglaucomatous participants were about 1298.84 RF; SD: 241.59 95% CI: 1201.26–1396.42 (*p* = 0.0002).

As recorded, the first line of antioxidant defense represented by total SOD activity in POAG patients (17.48 NU/ml; SD: 3,12 95% CI: 16,3–18,6) was decreased as compared with healthy controls (20,43 NU/ml; SD: 4,03 95% CI: 18,80–22,06) - Table 2. The differences reached statistical significance (*p* = 0.003). When SOD isoform activities were assessed separately significant diversification of examined groups were noted only in mitochondrial SOD2 (Mn-SOD) (glaucoma: 8.61 NU/ml; SD: 2.04 vs controls: 10.59 NU/ml; SD: 3.49. *p* = 0.048) – Table 2. The differences between compared groups in the mean cytosolic SOD1 (Cu,Zn-SOD) activity failed to reach significance (glaucoma: 8.87 NU/ml; SD: 2.44 vs controls: 9.84 NU/ml; SD: 2.20. *p* = 0.13).

No correlation between concentration of MDA (*p* = 0.29), LPS (*p* = 0.14), activity of total SOD (*p* = 0.4), Mn-SOD (*p* = 0.2), Cu,Zn-SOD (*p* = 0.99), TOS (*p* = 0.45), TAC (*p* = 0.23) and N95 amplitude was found.

Total oxidative state (TOS) showed intensification in the glaucoma group as compared with controls (22.81 μmol/l; SD: 26,44; 95% CI: 12,94–32,68 vs 8.08 μmol/l; SD: 5,82; 95% CI: 4,71–9,35; *p* = 0.016). Total antioxidant capacity (TAC) was comparable in examined patients (1.02 mmol/l; SD: 0,09; 95% CI: 0,98–1,06 in controls and 1.03 mmol/l; SD: 0,19; 95% CI: 0,96–1,10 in glaucoma, *p* = 0.31).

## Discussion

In this study, we found increased oxidative stress in patients with primary glaucoma. Changes in redox state were observed by enzymatic defense (SOD), accumulation of oxidative stress products (LPS and MDA) and total oxidant state.

Slightly different from previous experimental protocols, we designed this study model to select from participants scheduled for surgical procedures. Medical pre-operative evaluation was conducted in the hospital, which provided well-documented medical history of those involved in the study as compared with e.g., policlinic research model(s). Primary glaucoma is rather generalized than just an ocular disorder. Therefore, we believe, to examine primary glaucoma precisely, the general clinical condition of patients should be carefully studied.

We have included patients with documented visual field (VF) damage progression in the POAG group. Although performed, visual field analysis of participants was intentionally precluded. To evaluate glaucomatous damage in the course of oxidative stress precisely, in our study, we chose the transient pattern electroretinogram (PERG). The PERG is less depended on lens opacification than visual field examination. In glaucoma, both PERG amplitude reduction and implicit time increase have been reported in various studies, however the implicit time increase is relatively small and amplitude reduction have drawn the most interest. Results from experimental studies (primate model) indicate that PERG amplitude reduction precede the development of significant changes in the optic nerve head, and are related to the degree of cupping and nerve fiber loss, and are not diminished when IOP is reduced pharmacologically [17].

**Fig. 1:**
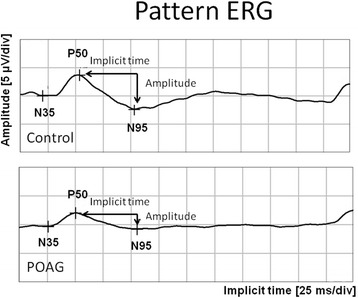
Pattern ERG representative for subjects from control and POAG group. Lowered N95 amplitude in eye with glaucomatous neuropathy as compared with control. μV/div – microvolts per division; ms/div – milliseconds per division

Therefore, for patient comparisons and RGC vitality assessment PERG is more precise, and less dependent on physician interpretation. Optic nerve diseases preferentially affect N95 and its amplitude is decreased or absent in optic atrophy [18]. In this current study, no significant differences in the N95 implicit time between the study groups were found. The N95 amplitude was notably significantly lower in the POAG group than in the control group, which reflects advanced glaucomatous atrophic changes of the optic nerve. No correlation between the N95 amplitude and serum level of analyzed substances indicates that PERG does not reflect rapid local changes in ganglion cells’ activity in the presence of oxidative stress (Fig. 1).

Mittag et al. demonstrated the developing changes in optic nerve atrophy experimentally in a rat glaucoma model. Their research suggests ERG responses begin to decline after 3 to 4 months of about 100% increase in IOP [19].

As described, glaucomatous pathological processes fluctuate over stable and progressive states [20]. Our glaucoma group patients were scheduled for surgery due to glaucomatous neuropathy exacerbation. Therefore, our examined patients were presenting in the active phase of neuropathy. The examined group of patients presented with oscillating IOP around is upper limit and comparable among themselves.

We decided to include patients presenting with cataract in both the examined group and the control. Oxidative stress has long been involved in the pathogenesis of cataract [21]. As suggested by Nucci et al. [6], comparing the age-matched and cataract-matched groups is more precise in the context of oxidative stress.

Noteworthy to mention, we sampled one tube of blood serum for the measurement of redox status, incidentally selecting an instant of the glaucomatous process. Thus, we should include this determination as a screening test. On the other hand, we included this eye in the research that revealed progressive neuropathy; this was assessed with visual field and electrophysiology over the last 6 months.

We examined eyes with IOP less or equal to 23 mmHg over the last 6 months. Thus, we assume that primary oxidative stress delivered with blood has a remarkable impact on the glaucomatous process than topical oxidative stress: that could be produced by intraocular pressure ≤23 mmHg.

We intentionally analyzed only one eye per patient because the second eye in many cases was after intrabulbar surgery (drainage or/and cataract) which could have additional impacts on the glaucomatous process in this eye.

The serum concentration of lipofuscin in POAG was significantly higher as compared with controls. Considered together with the research of Fernandez de Castro et al. [8], when lipofuscin concentration was recorded proportionally higher in the optic nerve of glaucomatous subjects, this suggests the age-related lipopigment involvement in the exacerbation of a glaucomatous neuropathy in the course of age-related POAG.

Significant increase in MDA serum concentration (almost two fold) corresponds with results as a marker of oxidation degradation products evaluated in serum [22], aqueous humor [23] and both, serum and humor [6], erythrocytes [5] and even in the optic nerve head [24]. This supports that systemic pathological processes are reflected in the glaucomatous eye, both by increasing IOP and exacerbation of RGC death. It should be mentioned that lipid metabolism has an impact on the amount of MDA formation, thus we have included patients with comparable body mass index into our study.

Activity of the total superoxide dismutase in our research model was significantly decreased in glaucomatous patients and corresponds with observations of Engin et al. [22]. Presumably, this decrease results from the depletion of the antioxidant defense system due to long exposure of oxidative stress. Glaucomatous patients in our research presented with exacerbated oxidative stress as indicated by TOS differences between groups. When focused on isoenzymes separately, only SOD2 presented significant deficiency. Emerging evidence suggests that SOD2 (mitochondrial), not SOD1 has a protective role against neuronal cell death induced by glutamate excitotoxicity and oxidative stress [24].

Finally, we collated our results with other previous observation regarding accumulation of MD, LPS and tSOD activity in serum, aqueous humor, lamina cribrosa and optic nerve of patients with POAG (Table 3).

**Table 3:** Previously reported LPS, MDA accumulation and tSOD activity in POAG group as compared with non-glaucoma patients

	Increased	Decreased	Not affected
LPS accumulation	SERUM^present study^ LAMINA CRIBROSA [25] OPTIC NERVE [8]	Not reported	Not reported
MDA accumulation	SERUM ^present study +^ [22, 26] AH^b^ [23, 27] SERUM + AH [6] LAMINA CRIBROSA [28]^a^	Not reported	Not reported
tSOD activity	SERUM [26] AH [10, 27, 29]	SERUM^present study +^ [22] AH [30]	Not reported

^a^unclear type of glaucoma

^b^ *AH* aqueous humour

In our study, the antioxidative reserves (TAC) were comparable with controls and oxidative stress. TOS was higher in glaucomatous participants. It should be recognized that only some antioxidative enzymes could play a key role in a glaucomatous pathology.

Finally, the biggest limitations of present study should be mentioned. This study model made it impossible to compare progressing with nonprogressing glaucomatous subjects in the context of oxidative stress. For such a comparison, a long-term prospective study with detailed monitoring of systemic and topic conditions is most suitable. Examined groups are small. However, the results are encouraging enough for designing a larger study. Groups were not genetically investigated.

## Conclusion

Systemic redox balance of cataract patients was significantly altered in the course of glaucoma.

## Abbreviations

AM: Ante meridiem
BMI: Body mass index
CBC: Complete blood count
CI: Confidence interval
CRP: C-reactive protein
Cu,Zn-SOD (SOD1): Cystolic superoxide dismutase
ERG: Electroretinogram
IOP: Intraocular pressure
KCN: Potassium Cyanide
LPS: Lipofuscine
MDA: Malondialdehyde
Mn-SOD (SOD2): Mitochondrial superoxide dismutase
NU: Nitric unit
PERG: Pattern electroretinogram
POAG: Primary open-angle glaucoma
RF: Relative fluorescence lipid extract
RGC: Retinal ganglion cell
ROS: Reactive oxygen species
RPM: Revolutions per minute
SD: Standard deviation
SOD: Superoxide dismutase
TAC: Total antioxidant capacity
TBARS: 2-thiobarbituric acid-reactive substance
TOS: Total oxidant state
VF: Visual field
